# Genetic Conservation and Population Management of Non‐Human Primates: Parentage Determination Using Seven Microsatellite‐Based Multiplexes

**DOI:** 10.1002/ece3.71216

**Published:** 2025-04-07

**Authors:** Natasja G. de Groot, Annemiek J. M. de Vos‐Rouweler, Corrine M. C. Heijmans, Annet Louwerse, Jorg J. M. Massen, Jan A. M. Langermans, Ronald E. Bontrop, Jesse Bruijnesteijn

**Affiliations:** ^1^ Comparative Genetics and Refinement Biomedical Primate Research Centre Rijswijk GJ the Netherlands; ^2^ Animal Science Department Biomedical Primate Research Centre Rijswijk GJ the Netherlands; ^3^ Animal Behaviour and Cognition, Department of Biology Utrecht University Utrecht CH the Netherlands; ^4^ Population Health Sciences, Unit Animals in Science and Society, Faculty of Veterinary Medicine Utrecht University Utrecht CM the Netherlands; ^5^ Theoretical Biology and Bioinformatics Utrecht University Utrecht CH the Netherlands

**Keywords:** apes, molecular genetics, NHP, non‐invasive, Old World monkey, parentage

## Abstract

Conservation of non‐human primates receives much attention, with nearly 350 of the more than 520 recorded primate species classified as threatened. To conduct effective population management, monitoring genetic diversity within species is of key importance, as it can offer insights into the levels of inbreeding within groups or populations. To examine kinship within the macaque breeding groups housed at the Biomedical Primate Research Centre, located in Rijswijk, The Netherlands, we have developed seven microsatellite‐based multiplexes for parentage analysis. These multiplexes comprise a unique set of 23 short tandem repeats (STR) distributed across 15 chromosomes. Extensive validation has been conducted across 2217 Indian rhesus ( *Macaca mulatta* ) and 759 long‐tailed macaques ( *M. fascicularis* ), demonstrating that these STR markers are highly polymorphic and segregate. Most markers exhibit a polymorphic information content (PIC) value above 0.5, illustrating that they are highly informative and valuable in providing us with a reliable parentage determination. Beyond macaques, we manifested that the multiplexes are also suitable for addressing parentage issues in apes and other Old World monkey species. Furthermore, this assay works on DNA isolated from both invasive and non‐invasive derived material (e.g., hair follicles and potentially feces). Thus, we present here seven validated multiplexes suitable for parentage analysis in apes and Old World monkey species. These multiplexes support future colony management objectives for various captive populations and, given the applicability of non‐invasive techniques, could also be valuable for monitoring free‐ranging primate populations.

## Graphical abstract

We have developed and extensively validated seven microsatellite‐based multiplexes consisting of 23 STR markers distributed across 15 chromosomes for parentage analysis in apes and Old World monkey species. DNA isolated from both invasive and non‐invasively collected biomaterials can be used. This approach can benefit future conservation and population management strategies for both captive and free‐ranging primate populations.

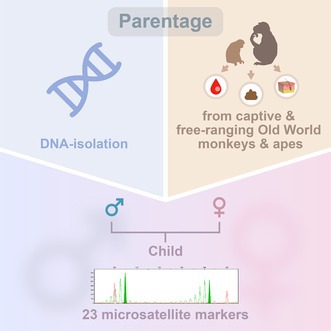

**Abbreviations**
AAALAC: American Association for Accreditation of Laboratory Animal Care
BPRC: Biomedical Primate Research Centre
EDTA: ethylenediamine tetraacetic acid
HW: Hardy–Weinberg equilibrium
LOD: logarithm of the odds
MHC: major histocompatibility complex
PCR: polymerase chain reaction
PIC: polymorphic information content
SNP: single nucleotide polymorphism
STR: short tandem repeat

## 1 Introduction

Worldwide, there are over 520 recognized non‐human primate (NHP) species, which have their natural habitat in parts of South America, Africa, South Asia, and Southeast Asia (Estrada et al. 2022). Approximately 67% of these species are listed as threatened on the “Red List of Endangered Species” of the International Union for the Conservation of Nature (IUCN 2023, https://www.iucnredlist.org). Their vulnerability is largely driven by habitat destruction and the decrease in availability of natural resources due to human interference (Chapman and Peres 2021; Fernandez et al. 2022). Consequently, populations become fragmented, potentially impacting population size, genetic diversity, and overall fitness of a population (Bergl et al. 2008; Kuang et al. 2020; Molina‐Vacas et al. 2023; Schwitzer et al. 2014; Wang et al. 2017). The emergence of smaller isolated populations also increases the risk of inbreeding (Guo et al. 2020; Williams et al. 2020), which may ultimately lead to extinction of a species. To address these issues, conservation management strategies aimed at supporting the long‐term survival and well‐being of NHPs have received much attention. This focus has resulted in an increase in available information since the beginning of the 20‐first century showing the significant impact of habitat destruction, the decrease of availability of natural resources, and climate change on the fitness of NHP populations (Chapman and Peres 2021; Fernandez et al. 2022). Whereas the impact is in general reported as a decrease in population or individual numbers of a particular species, little is known about how this effects genetic diversity in the remaining populations.

In addition to conservation efforts focused on preserving NHP in their natural habitats, numerous global organizations and zoos are actively involved in initiatives aimed at ensuring the welfare and long‐term prospects of animals held in captivity. However, animals kept in zoological institutions often live in small groups, enhancing the likelihood of inbreeding. To address this concern, breeding and zoo exchange programs have been established (e.g., see https://www.eaza.net/eep‐pages/), but their effectiveness depends on various factors, including successful social integration of individuals into new groups and the capability to reproduce. Furthermore, certain species are represented by only a few animals held in captivity, which might impose limitations on potential conservation strategies. A key aspect in population management is a thorough understanding of kinship relationships within a group of animals, as breeding among closely related individuals can have genetic consequences and increase the likelihood of homozygosity. Accordingly, reduced genetic diversity can increase susceptibility to infectious diseases and parasite burdens (Antunes et al. 1998; McKnight et al. 2017; Reed and Frankham 2003; Schwensow et al. 2007), potentially exacerbating population decline.

For decades, microsatellite markers, also referred to as short tandem repeats (STR), have been developed and utilized for parentage analyses (Flanagan and Jones 2019). These molecular markers enable the measurement of multi‐locus heterozygosity at different chromosomes, providing insights into the genetic diversity within a population and offering an estimate of the extent of inbreeding levels. Advancements in next‐generation sequencing techniques have facilitated the implementation of single nucleotide polymorphisms (SNP) and SNP‐arrays in these types of analyses. Both microsatellite‐ and SNP‐studies have demonstrated their effectiveness in the context of parentage determination (Flanagan and Jones 2019). However, the choice between either one of these methods depends on various factors, including the specific characteristics of the studied population and the type of available samples (Stadele and Vigilant 2016). For instance, in situations involving non‐model species and free‐ranging populations, where obtaining predominantly low‐quality DNA is the norm, microsatellite analyses often emerge as the most suitable option. Additionally, the application of microsatellite markers may offer cost‐effective advantages and require less intensive bioinformatics for data analysis as compared to SNP‐based approaches.

The Biomedical Primate Research Centre (BPRC) houses self‐sustaining breeding colonies of Indian rhesus macaques ( *Macaca mulatta* ) and long‐tailed macaques ( *Macaca fascicularis* ), also referred to as crab‐eating or cynomolgus macaque, which were established decades ago. These colonies are organized into various multigenerational social groups, with a breeding strategy focused on maintaining genetic diversity by using the major histocompatibility complex (MHC) as a marker. Currently, there are approximately 24 rhesus and eight long‐tailed macaque groups, each consisting of 10 to 50 individuals. In the distant past, we determined kinship by monitoring the segregation of a few polymorphic MHC markers, mapped to a single chromosome and detected by alloantisera (Bontrop et al. 1995). Later, sequencing analysis was applied to determine these and various other polymorphic *MHC* genes to define kinship (Doxiadis et al. 2013; Otting et al.  2000, 2002, 2005, 2007). To enhance colony management, we have developed seven microsatellite‐based multiplexes targeting 15 chromosomes that allow a rigid molecular identification of parentage within these groups. Furthermore, we demonstrate that these multiplexes are also applicable to apes and other Old World monkey species. They can utilize DNA sourced from blood, as well as extracted from non‐invasively collected biomaterials such as hair follicles and potentially feces. These seven multiplexes are suitable for molecular‐level parentage monitoring across both free‐ranging primate populations and those living in for example, zoos and sanctuaries, to facilitate more comprehensive colony management strategies.

## 2 Materials and Methods

### 2.1 Samples and Genomic DNA Isolation

The BPRC (Rijswijk, the Netherlands) is an AAALAC‐accredited facility that houses self‐sustaining breeding colonies of rhesus and long‐tailed macaques. The rhesus macaques are of Indian origin, and this colony was founded in the 1970s with over 140 animals (Doxiadis et al. 2013). The long‐tailed macaques originate from regions north and south of the isthmus of Kra, as well as from the Indonesian and Malaysian islands (de Groot et al. 2022). The orignal cohort, kept at the University of Utrecht (Otting et al. 2009), was founded in 1965 with a small number of animals and was expanded in 1980 with approximately 20 additional animals to form stable groups. The cohort grew to 135 animals, which were transferred to the BPRC facilities in 2009 (Otting et al. 2009). Subsequently, between 2011 and 2016, 84 long‐tailed macaques, originating from Chinese and other European breeding facilities, were introduced into the original cohort to maintain genetic diversity (de Groot et al. 2022). Both macaque breeding colonies are divided into multiple naturalistic social groups that typically contain various matrilines, including adult females and their offspring, along with a single non‐natal adult male. To prevent inbreeding and conflicts, males above 3 years of age are dispersed from the group whenever feasible. During annual health check‐ups, in accordance with the Dutch regulations of animal welfare, EDTA‐blood samples are collected from the animals for the extraction of genomic DNA (gDNA) using a standard salting‐out procedure. Additionally, hair follicle samples from stillborn animals or young infants that die shortly after birth are collected, from which DNA is extracted using the Puregene DNA purification kit (Qiagen), following the manufacturer's recommendations.

In the past, BPRC housed a large pedigreed population of West African chimpanzees ( *Pan troglodytes verus* ) that started with 35 founder animals and expanded to over 200 animals. Following the prohibition on using chimpanzees in biomedical research in 2003 by the Dutch government, all animals were relocated to European zoos and sanctuaries. For most of these chimpanzees, DNA samples are stored in BPRC's Biobank. Safaripark Beekse Bergen in Hilvarenbeek, The Netherlands, is home to a group of chimpanzees that originated from the BPRC. In 2013, three hair follicle samples collected from two newborn chimpanzees within this group were sent to BPRC for paternity determination, and DNA was isolated as described above. These chimpanzee samples were collected in accordance with the Dutch regulations of animal welfare.

Fecal samples from Moloch gibbons ( *Hylobates moloch* ), also known as Silvery Javan gibbons, residing at the Port Lympne and Howletts zoological institutions in the UK, were collected for an initial MHC‐typing study (de Groot et al. 2017). For these non‐invasively obtained samples, no animal care and use authorization was required. Briefly, DNA was extracted from the frozen fecal samples using the QIAamp DNA stool Mini Kit (Qiagen) following the Stool Larger Volumes protocol.

In January 2016, a cohort of Hamadryas baboons ( *Papio hamadryas* ) housed in WILDLANDS Adventure Zoo in Emmen, the Netherlands, underwent health check‐ups before the relocation from the old zoo facilities to their new accommodation. This cohort has been a closed population for decades, and it is assumed that all baboons in the group are related to some extent. EDTA‐blood samples from this cohort were collected during the health check‐ups in accordance with the Dutch regulations of animal welfare and used to extract gDNA using a standard salting‐out procedure or the AllPrep DNA/RNA kit (Qiagen).

### 2.2 Microsatellite Markers Used for Parentage Analyses

The microsatellite markers included in the parentage analysis comprise: D1S548, D2S169, D3S1768, D4S2365, D5S820, D5S1457, D6S276, D6S291, D6S1691, D7S513, D7S794, D8S1106, D10S1412, D11S925, D12S67, D13S765, D14S306, D18S72, DXS2506, MFGT18, MFGT21, MFGT22, and MFGT27. Table S1 provides additional relevant information for these markers, such as chromosome location, primer sequences, and labeling dyes (Table S1).

### 2.3 Amplification and Genotyping of Microsatellite Markers

The polymerase chain reaction (PCR) mixture (17.5 μL) contains 2.5 μL gDNA (concentration between 25 and 100 ng/μL), 3.2 μL of 10 μM STR‐multiplex primer mix stock solution (Table S1), 1× PCR buffer, 5 mM MgCl_2_, 0.2 mM of each deoxyribonucleoside triphosphate (dNTP), and 0.04 Units *Taq* polymerase (Invitrogen). For multiplex primer mix MuA, MuBC, MuD, MuE, MuF2, and MHC‐STR set‐2, the amplification started with 4 min at 94°C, followed by 5 cycles each consisting of 1 min at 94°C, 45 s at 58°C, and 45 s at 72°C, and then 25 cycles each consisting of 45 s at 94°C, 30 s at 58°C, and 45 s at 72°C, and a final extension of 30 min at 72°C. For multiplex primer mix MuF1, the annealing temperature was set to 60°C instead of 58°C. For genotyping, 3 μL of PCR product was mixed with 15 μL of a standard mix, containing 15 μL H_2_O and 0.5 μL ROX350 or ROX500 size standard (Applied Biosystems). After a heat shock for 2 min at 94°C, the samples were run on an ABI 3100, 3130*xl*, or 3500*xl* genetic analyzer (Applied Biosystems). At least one negative control sample and four historical controls are included per 96‐well plate.

### 2.4 Data Analysis

The sizes of the PCR amplified fragments are determined with the GeneMapper software, version 5.0 (Applied Biosystems).

Allele frequency analysis was performed with the Cervus software package (http://www.fieldgenetics.com), version 3.0.7 (Kalinowski et al. 2007), and is defined as the relative frequency of alleles at a particular locus in a population. In addition, the Cervus software package, which is widely applied for parentage analysis in plant and animal populations, was used to calculate the paternity inference likelihood ratio. This approach accounts for cases where the genotypes of either parent or of the offspring are incomplete or inaccurately assessed. The program generates a table providing the number of loci typed, pair loci compared, pair loci mismatching, pair LOD score (logarithm of the odds), pair delta, and pair confidence. In addition, the same parameters are calculated for the offspring taken both parents into consideration: trio loci compared, trio loci mismatching, trio LOD score, trio delta, and trio confidence.

### 2.5 Statistics

For the 23 microsatellite markers, different parameters were computed using the Cervus software package. This involved: (1) the number of alleles; (2) number of typed animals; (3) observed heterozygosity (Ho), which is a measure representing the proportion of individuals in the population that are heterozygous at a given locus calculated based on known genotype frequencies in the population; (4) expected heterozygosity (He), which indicates the expected fraction of heterozygotes in the population under the Hardy–Weinberg model computed using known allele frequencies; (5) polymorphic information content (PIC), which assesses a marker's ability to detect polymorphism and expresses the marker's usefulness for diversity analysis. According to the criteria of Botstein et al. (Botstein et al. 1980), PIC > 0.5 is highly informative, 0.25 < PIC < 0.5 is moderately informative, and PIC < 0.25 is slightly informative; (6) average non‐exclusion probability for one candidate parent (NE‐1P); (7) average non‐exclusion probability for one candidate parent, given the genotype of a known parent of the opposite sex (NE‐2P); (8) significance of a deviation from the Hardy–Weinberg equilibrium, including the Bonferroni correction (HW); and (9) estimated null allele frequency (F(Null)). A null allele can occur due to sequence amplification failure because of a mismatch in primer binding sequence. Cervus recommends a null allele frequency level lower than 0.05.

## 3 Results

### 3.1 Development of Seven Microsatellite‐Based Multiplexes for Parentage Analysis in NHP

**FIGURE 1:**
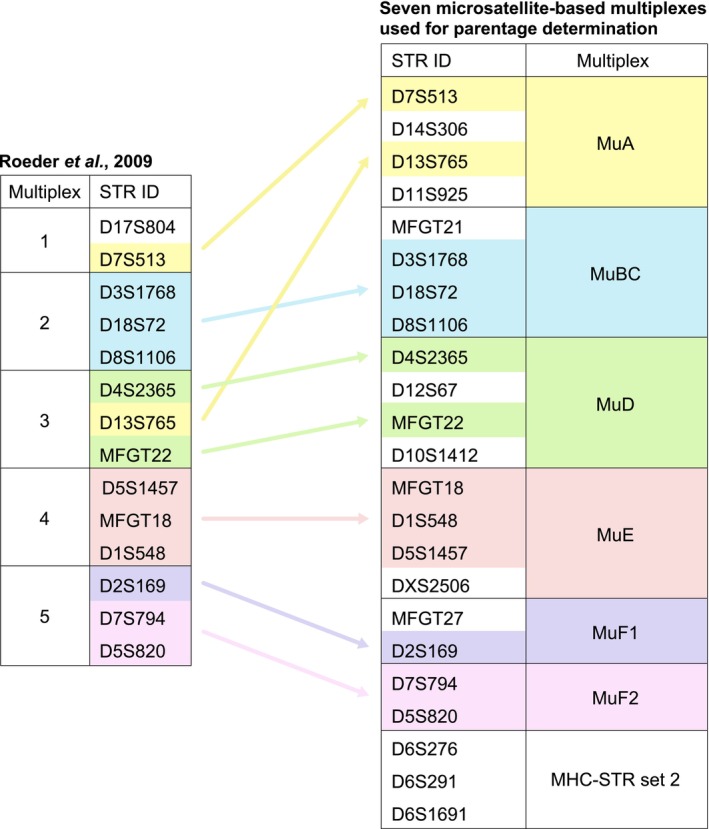
Overview of the five microsatellite multiplexes previously published by the INPRIMAT consortium (left) (Roeder et al. 2009) compared to our current extended and refined microsatellite multiplexes (right). The arrows and color codes depict corresponding short tandem repeat (STR) identification (ID) numbers between both panels. Details regarding the various microsatellite markers included in the multiplexes are provided in (Data S1; Table S1).

Most of the markers incorporated in our microsatellite‐based multiplexes were initially described and used to monitor diversity in the human population and subsequently tested for their cross‐species amplification and polymorphism in NHP (de Groot, Heijmans, et al. 2008; Penedo et al. 2005; Roeder et al. 2009). Based on the set of microsatellite data provided by the INPRIMAT consortium (Roeder et al. 2009), we extended, refined, and optimized the five previously published multiplexes (Multiplex 1–5). This resulted in the present seven multiplexes for parentage determination (Figure 1; Table S1).

During the development phase, a range of microsatellite markers was incorporated and tested in various combinations to enhance the five INPRIMAT multiplexes, which in total comprised 14 markers distributed across 11 chromosomes. Marker D17S804, initially featured in Multiplex 1 (Figure 1), was excluded because of its limited levels of polymorphism detected in the rhesus and long‐tailed macaque populations housed at BPRC. Ultimately, 10 additional microsatellite markers, namely D6S267, D6S291, D6S1691, D10S1412, D11S925, D12S67, D14S306, DXS2506, MFGT21, and MFGT27, were identified as informative and integrated into different multiplexes (Figure 1). The composition of microsatellite markers within each multiplex was tailored to achieve optimal performance. The resulting system now comprises seven multiplexes, designated as MuA to MuF2 and MHC‐STR set‐2, collectively encompassing 23 microsatellite markers distributed across 15 chromosomes (Table S1).

### 3.2 Validation of the Seven Microsatellite‐Based Multiplexes in Two Macaque Cohorts

The rhesus and long‐tailed macaques housed at the facilities of the BPRC were instrumental in the development and validation of the seven microsatellite‐based multiplexes.

**FIGURE 2:**
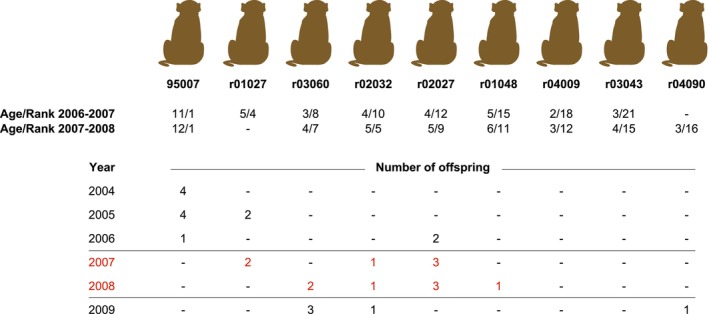
Overview of the offspring distribution produced by the different males in a breeding group of rhesus macaques. For each animal, their age and rank position within the breeding group are provided, utilizing data collected during the two observational periods, 2006–2007 and 2007–2008 (Massen et al. 2012). Animal r01027 was removed from the breeding group in 2007–2008 due to his age and rank. Highlighted in red is the period between 2007 and 2008, during which animal 95007 maintained his status as alpha male, but offspring were produced by other males in the group.

The major development of the seven multiplexes was initiated through a study involving a multimale‐multifemale breeding group of rhesus macaques that focused on understanding male mating tactics (Massen et al. 2012). Rhesus macaques are seasonal breeders, with mating typically occurring from October through December, followed by females giving birth during the spring. During a 2‐year observational period, animal 95007 was recognized as the alpha male, while the hierarchy of the other males evolved (Figure 2). All offspring born in this group during the specified period, as well as those born in the years 2004–2006 and 2009, were included in parentage analysis. At that time, the test involved 20 microsatellite markers, 13 of which are incorporated in the current seven multiplexes (Massen et al. 2012). Parentage analyses revealed that the alpha male from 2006 to 2008 did not produce any offspring (Figure 2).

**FIGURE 3:**
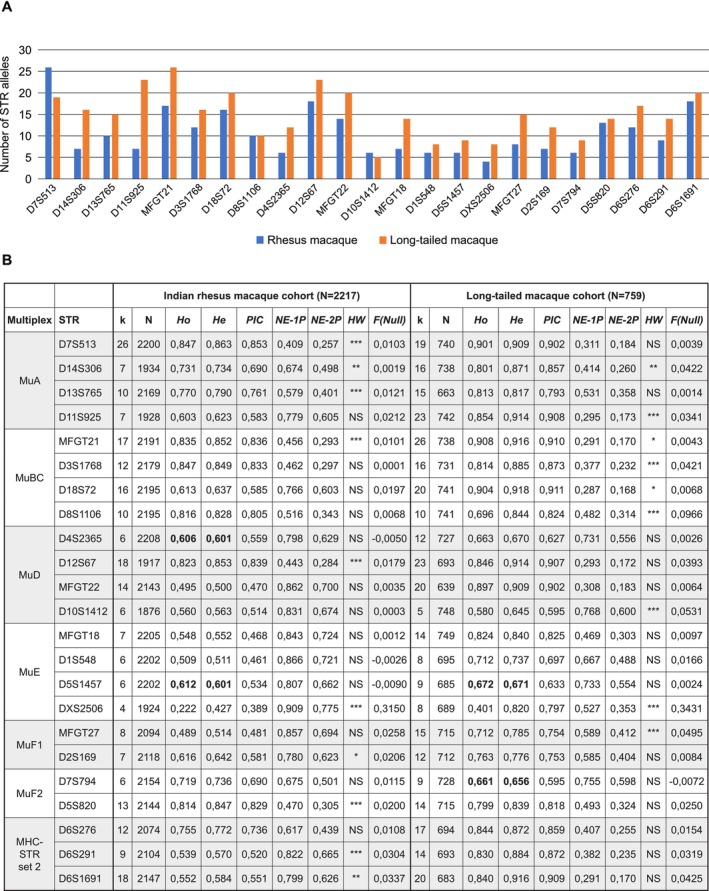
Overview of the STR allele distribution (A) and various calculated parameters (B) of the microsatellite markers included in the seven multiplexes for the rhesus and long‐tailed macaque cohorts. (B) Number of alleles (*k*), number of typed animals (*N*), observed (*Ho*) and expected (*He*) heterozygosity, polymorphic information content (*PIC*), average non‐exclusion probability for one candidate parent (NE‐1P), average non‐exclusion probability for one candidate parent given the genotype of a known parent of the opposite sex (NE‐2P), significance of deviation from Hardy–Weinberg equilibrium (HW; NS not significant and *, **, *** significance at the 5%, 1%, 0.1% level, respectively), and estimated null allele frequency (F(Null) are provided. Bold printed numbers highlight microsatellite markers for which a slightly higher Ho is observed as compared to He.

As of 2011, the seven multiplexes have been utilized for parentage analysis of all newborn animals, stillborn animals, and young infants that die shortly after birth in our macaque breeding groups. Furthermore, DNA samples collected from all other animals in the two cohorts, as well as historically collected DNA samples, were retrospectively analyzed for parentage determination. The current STR fragment analysis results encompass 2,217 rhesus and 759 long‐tailed macaques (data up to December 15, 2023). All 23 markers included in the seven multiplexes exhibit high polymorphism within the two cohorts (Figure 3). In the rhesus and long‐tailed macaque breeding groups, the number of alleles per marker ranges from four to 26 (Figure 3A), with averages of 10.7 and 15 alleles, respectively. The observed heterozygosity (*Ho*) ranges from 0.222 (DXS2506) to 0.847 (D3S176 and D7S513) in rhesus macaques and from 0.401 (DXS2506) to 0.908 (MFGT21) in long‐tailed macaques. Expected heterozygosity (*He*) ranges from 0.427 (DXS2506) to 0.863 (D7S513) in rhesus macaques and from 0,645 (D10S1412) to 0.918 (D18S72) in long‐tailed macaques (Figure 3B). Most markers have a polymorphic information content (PIC) value > 0.5, indicating that they are highly informative (Figure 3B). Thirteen and fourteen markers in the rhesus and long‐tailed macaque cohorts, respectively, were found to display allele frequency distributions conforming to Hardy Weinberg (HW) expectations. Ten markers in the rhesus macaque cohort and nine markers in the long‐tailed macaque cohort deviate from HW expectations at varying levels of significance (Figure 3B; Table S3). This deviation is most likely caused by our breeding strategy, which is based on non‐random mating. More in detail, we select alpha males based on their variation in the polymorphic *MHC* genes, ensuring genetic differences from the females to which the male is introduced. The null allele frequency is, for most markers, in accordance with the recommendations of Cervus software, with exceptions for DXS2506 in both cohorts and D8S1106 in the long‐tailed macaque cohort (Figure 3B).

In conclusion, the calculated parameters for the 23 microsatellite markers in the large sample of rhesus and long‐tailed macaques demonstrate that the seven multiplexes comprise informative STR markers capable of accurately performing parentage analysis.

### 3.3 Parentage Determination in Rhesus and Long‐Tailed Macaque Cohorts Using the Microsatellite‐Based Multiplexes

**FIGURE 4:**
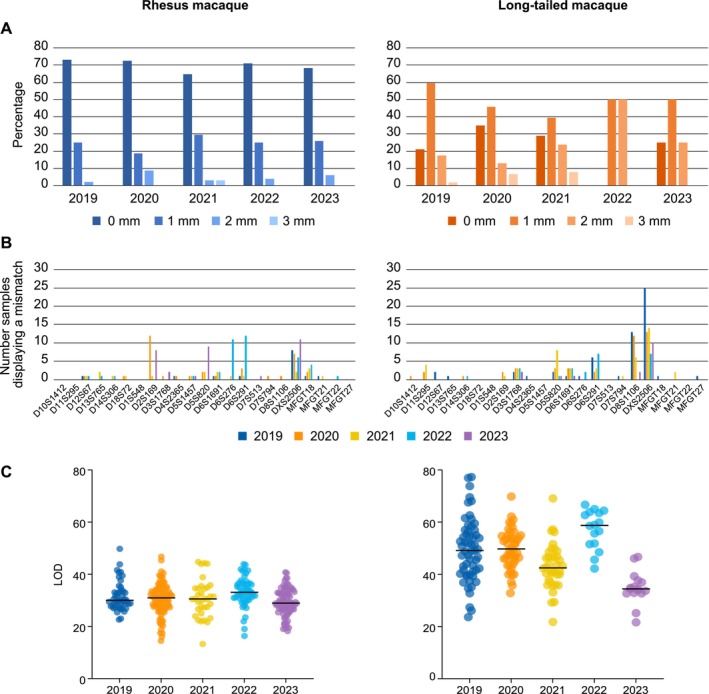
Bar charts (A, B) and LOD metrics (C) on parentage analysis in BPRC's rhesus (left panel) and long‐tailed (right panel) macaque cohorts for the years 2019 to 2023. (A) The panels display the percentage of offspring for which the parents were assigned with 0, 1, 2, and 3 mismatches (mm) based on trio loci mismatching. (B) The panels show the number of samples that displayed a mismatch at a corresponding microsatellite marker. (C) The panels present a scatter plot of the trio LOD scores calculated for each offspring, with the median LOD score indicated.

To assign parents to their offspring, we analyzed the fragment length data of the 23 STR markers within the multiplexes for the offspring and their potential parents using Cervus. Here we present the results of the parentage analysis conducted over the past 5 years, from 2019 to 2023, on a total of 321 rhesus (296 blood and 25 hair samples) and 173 long‐tailed macaques (166 blood and 7 hair samples). For these offspring, parents were assigned with up to three mismatches based on trio loci mismatching. In the rhesus macaque samples, parentage was successfully assigned without mismatches in 226 (~70%) of the cases (Figure 4A; Table S2A). Parentage was assigned with one mismatch in 76 samples (~25% on average), and with two mismatches in 18 samples (~5% on average). One hair sample was assigned with three mismatches. Over the 5‐year period, approximately a quarter of the mismatches were attributed to allelic dropout at marker DXS2506 (Figure 4B). Occasionally, markers D2S169, D5S820, D6S276, and D6S291 may show a higher level of allelic dropout (Figure 4B), which contributed to the mismatches observed. In the long‐tailed macaque samples, on average, parentage was assigned with no mismatches in approximately 22% of the cases (43 samples), with one mismatch in about 50% (86 samples), and with two mismatches in roughly 26% of the cases (37 samples) (Figure 4A; Table S2A). Here, an elevated proportion of mismatches (37.5%) was similarly attributed to allelic dropout at marker DXS2506 (Figure 4B). This resulted in a higher frequency of samples defined with one mismatch rather than no mismatches in long‐tailed macaques, compared to the rhesus macaque samples. Furthermore, occasional higher levels of allelic dropouts were observed for markers D8S1106, D5S820, and D6S291, potentially due to technical issues. Seven long‐tailed macaque samples, including one hair sample, were assigned with three mismatches. The reliability of the parentage assignments, estimated by the log likelihood ratio test (LOD), ranged from 13.30 to 49.75 (total mean ± SEM, 30.79 ± 0.33) for rhesus macaques and from 21.64 to 77.32 (total mean ± SEM, 47.54 ± 0.82) for long‐tailed macaques (Figure 4C; Table S2B). These positive LOD scores imply a high probability that the assigned candidate parents are the true parents.

Next, we provide some examples of paternity and maternity inquiries that may arise in newborns that are resolvable using the parentage analysis results. In most cases, we confirm the parentage assignments made by the animal caretakers, drawing upon their experience. However, our analyses have also demonstrated that male dominance does not always correspond to mating access and reproductive success (Massen et al. 2012). Within breeding groups, conception sometimes occur among closely related individuals, such as siblings or half siblings. Occasionally, we observe very young females giving birth, some as young as 3 years old or even younger. This could potentially be the result of inbreeding, with the alpha male of the group being the father of the female in question. However, in most cases where a young rhesus macaque female gave birth, parentage typing revealed that conception occurred with similarly aged males rather than with the alpha male mating with his daughters. We observed that these young males are, in most cases, offspring of the previous alpha male of the group, thereby excluding inbreeding. Between 2011 and 2023, a total of 1498 offspring were born in the rhesus macaque cohort. Among these, 20 animals were identified with an inbreeding coefficient of 0.25 or higher, resulting from conception occurring between father and daughter, mother and son, or siblings (Table 1). The percentage of inbreeding per year fluctuates between 0% and 4.64%, with an average of 1.25% of the total births in these 13 years. In the long‐tailed macaque cohort during the same period, a total of 374 births were recorded, among which five offspring exhibited an inbreeding coefficient of 0.25 or higher (Table 1). This accounts for an average inbreeding rate of 1.18% of the total births within the specified time frame, which is similar to that observed in the larger rhesus macaque cohort.

**TABLE 1:** Overview of the total number of births each year from 2011 to 2023 in the rhesus and long‐tailed macaque cohorts, with the number of offspring having an inbreeding coefficient of 0.25 or higher indicated for each year. The last column displays the annual percentage of inbreeding.

Year	Total # of births	Conception between			% Inbreeding/year
		Father/daughter	Mother/son	Siblings	
Rhesus macaque cohort					
2011	148	—	1	—	0,67
2012	161	—	1	—	0.62
2013	177	2	—	—	1.13
2014	175	1	1	1	1.71
2015	151	4	3	—	4.64
2016	105	—	1	—	0.95
2017	144	1	—	—	0.69
2018	55	2	—	—	3.64
2019	37	—	—	—	0
2020	53	—	—	—	0
2021	102	—	—	—	0
2022	78	—	1	—	1.28
2023	112	—	1	—	0.89
Long‐tailed macaque cohort					
2011	18	1	—	—	5.55
2012	26	—	—	—	0
2013	24	—	—	—	0
2014	38	—	1	—	2.63
2015	38	—	—	—	0
2016	24	—	—	—	0
2017	41	—	—	—	0
2018	47	—	—	—	0
2019	42	—	3	—	7.14
2020	17	—	—	—	0
2021	17	—	—	—	0
2022	14	—	—	—	0
2023	28	—	—	—	0

**FIGURE 5:**
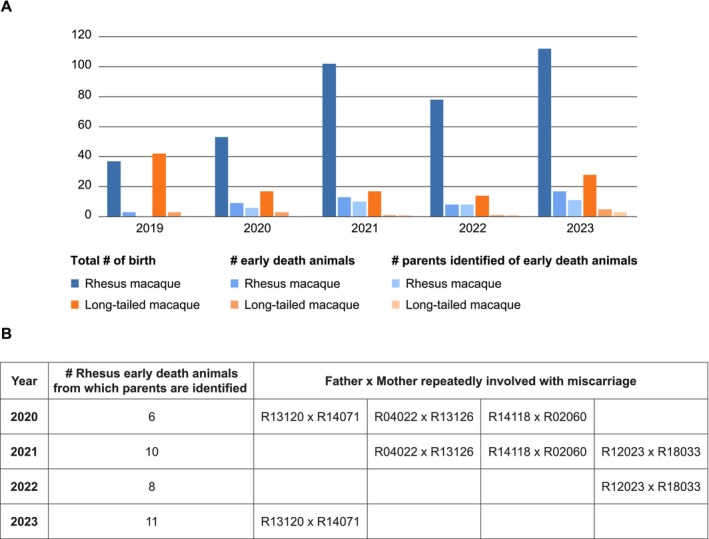
Information on birth rate, early death animals, and parent couples involved in recurrent miscarriages at the BPRC facilities from 2019 till 2023 (A) Bar chart showing the total number (#) of birth, # of early death animals, and # parents identified of early death animals for our rhesus and long‐tailed macaques (B) Overview of rhesus macaque parent couples that showed recurrent miscarriages in the period 2020 to 2023.

In addition to successful births, we also have a limited number of stillborn animals within each breeding season and animals that died shortly after birth (Figure 5A, referred to as early death animals). From a colony management perspective, it is important to monitor if specific parents are repeatedly involved in these incidents. In the 35 cases studied for rhesus macaques from 2020 to 2023, three parental couples were identified as having consecutive miscarriages (Figure 5B). One couple experienced miscarriages in both 2020 and 2023, while giving birth to two healthy babies in the intervening years (Figure 5B). Furthermore, three males, namely R04022, R14005, and R14118, were implicated in miscarriages with different females, accounting for six, four, and seven early death animals, respectively (data not shown). Of note, animals R04022 and R14118, with total offspring counts of 62 and 43, respectively, were also reproductively successful with most of the same females with whom they had experienced an early death birth.

Sometimes, caretakers observe a female carrying two babies, raising uncertainty about whether the female gave birth to twins or kidnapped a baby from another female. Parentage analysis helps to clarify these situations. Between 2011 and 2023, we conducted nine case studies. In six cases, we confirmed that they were twins. In two instances, the parentage analysis indicated that one of the babies was kidnapped, and in one case, we could not confirm the twin situation due to insufficient quality of DNA extracted from the collected hair sample.

Moreover, the outcomes of the parentage analyses are instrumental for breeding managers, especially when they need to split up breeding groups. Ensuring that the correct offspring migrate with their respective mothers into a new breeding group is essential for stable group dynamics and maintaining accurate lineage records and colony management.

### 3.4 The Seven Microsatellite‐Based Multiplexes: Expanding Beyond Macaques and Invasively Collected Samples

**FIGURE 6:**
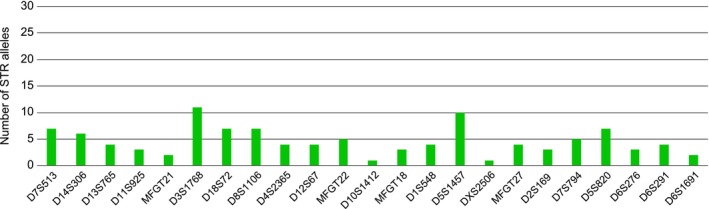
Bar chart illustrating the distribution of the number of STR alleles detected for each microsatellite marker included in the seven multiplexes for the Hamadryas baboon cohort.

**FIGURE 7:**
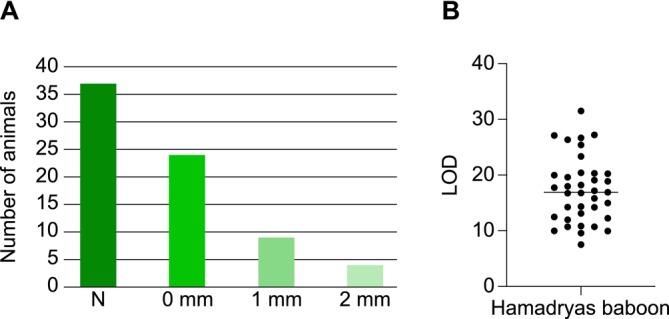
Bar chart (A) and LOD metrics (B) on the total number of Hamadryas baboon samples (*N*) for which parentage analysis could be conducted. (A) The number of offspring for which parents are assigned with 0, 1, and 2 mismatches (mm) based on trio loci mismatching are indicated. (B) Scatter plot of the trio LOD scores calculated per offspring plotted together with the median.

We have applied the seven microsatellite‐based multiplexes to establish parentage within a large cohort of zoo‐housed Hamadryas baboons. Baboons and macaques both belong to the Papionini tribe of the Old World monkey family and share a common ancestor approximately 6–8 million years ago (Steiper et al. 2004). Baboons have their natural habitat in equatorial Africa, whereas most of the macaque species are found on the Asian continent. Of the cohort, we have received 115 samples for analysis, with only limited background information on sex and age. All 23 microsatellite markers allowed amplification for these baboon samples. Two markers, D10S1412 and DXS2506, detected absence of polymorphism in this cohort. For the remaining markers, the number of alleles ranged from two to 11, with an average of 4.6 alleles (Figure 6; Table S4). Based on the PIC value, 11 markers were highly informative (PIC value > 0.5) and showed allele distributions conforming to HW expectations. Six markers were moderately informative (0.25 < PIC < 0.5), and three markers were classified as slightly informative (PIC < 0.25) (Table S4). In 37 cases, we were able to identify both parents with up to two mismatches, considering trio loci mismatching, and a LOD score ranging from 7.51 to 31,53 (mean ± SEM, 17.31 ± 0.96) (Figure 7; Table S2). For 18 animals, the parentage analyses identified only the mother, and for three animals, only the father. Because approximately half of the samples represented older animals (aged 9–36 years), most of which are assigned as father or mother in the groups of animals for which parents could be identified, additional sampling of potential parents is needed to further characterize this cohort for parentage analysis.

**FIGURE 8:**
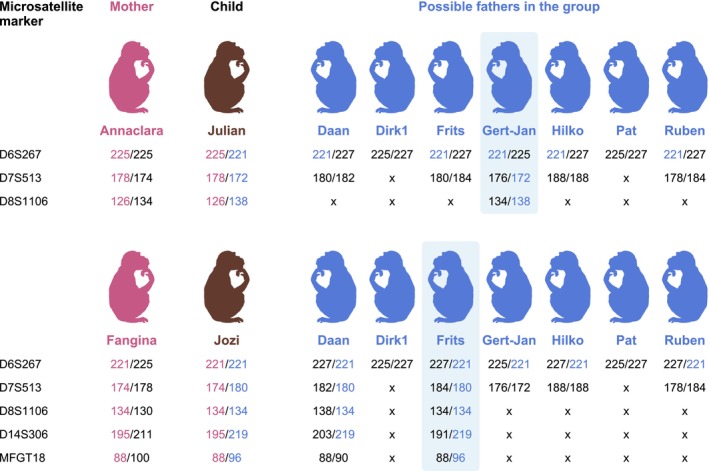
Graphical illustration of paternity analysis for two newborn chimpanzees, indicating the minimal number of microsatellite markers necessary to assign paternity. The results of the complete microsatellite dataset are provided in Table S5. The STR‐allele length indicated in pink is inherited from the mother, and the length indicated in blue is inherited from the father. When no corresponding STR‐allele length is found between the newborn chimpanzee and the possible father, an “x” is placed in the subsequent row to indicate that the individual in question could not be the father of the child.

At the BPRC, we previously housed a large cohort of chimpanzees for which DNA samples are stored in our biobank. These samples were instrumental in resolving two paternity questions within a group of chimpanzees originally from the BPRC cohort, currently residing in Safaripark Beekse Bergen. In 2013, DNA from the two mothers and seven potential fathers was retrieved from the BPRC biobank, together with DNA isolated from hair follicles collected from the two newborn chimpanzees subjected to parentage analysis using the seven multiplexes (Table S5). All markers of the seven multiplexes, except for MFGT27 and D5S280, showed successful amplification in the chimpanzee samples. Notably, at this stage, markers MFGT27, D2S169, D7S794, and D5S820 were still combined in one multiplex (MuF). However, our current approach split these into MuF1 and MuF2, each with a specific amplification temperature resulting in better amplification of the markers. Most markers exhibit polymorphism in the analyzed cohort, which included varying levels of familial relatedness (Table S5). One marker, MFGT21, displayed two alleles, while markers D3S1768, D6S291, D11S925, and DXS2596 lacked polymorphism. Among the 16 polymorphic markers, D6S276, D6S1691, D12S67, D10S1412, and D14S306 were newly introduced to the multiplexes, expanding upon those described by Roeder et al. in 2009. The polymorphic markers were instrumental in identifying Gert‐Jan as the father of Julian and Frits as the father of Jozi (Figure 8), from among the closely related males in the sample group who could have potentially been the father. For Julian, markers D6S267, D7S513, and D8S1106, and for Jozi, D6S267, D7S513, D8S1106, D14S306, and MFGT18 displayed a distinctive pattern (Figure 8).

**FIGURE 9:**
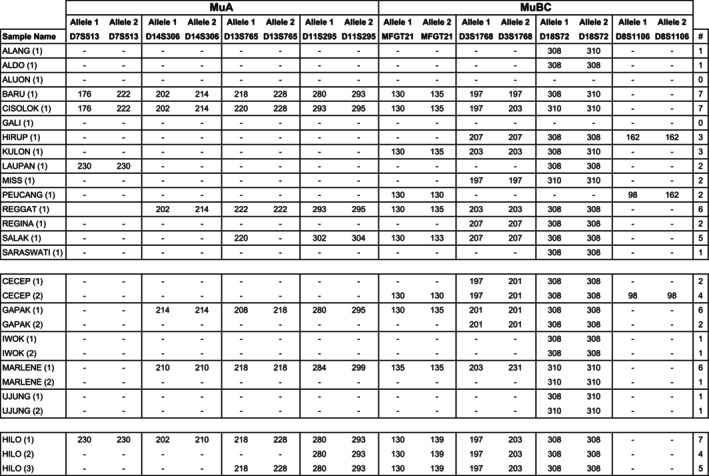
Overview of the length patterns detected for microsatellite markers in the multiplexes MuA and MuBC, using *Moloch* gibbon DNA samples isolated from feces. When a microsatellite marker is successfully amplified, the fragment lengths of the alleles are provided. In some instances, both alleles have identical fragment lengths, which may suggest that the gibbon in question is homozygous for the associated microsatellite marker. The last column summarizes the total number (#) of microsatellite markers amplified for a sample.

In addition, we tested a subset of the seven microsatellite‐based multiplexes for its applicability to DNA isolated from fecal samples collected from 21 zoo‐housed Moloch gibbons, a species of lesser apes. In this case, multiplexes MuA and MuBC were selected based on the type of microsatellite markers they contain, which demonstrated overall high allelic diversity in the rhesus and long‐tailed macaque cohorts investigated (Figure 3). Notably, the number of samples tested per individual was limited. For 15 individuals, a single DNA sample was analyzed, while for five individuals a duplicate sample was available, and one individual was examined in triplicate (Figure 9). All eight markers included in MuA and MuBC showed amplification in at least three of the fecal‐derived DNA samples, and different length patterns could be distinguished. However, the results also indicated that samples showed no amplification or only amplification of a subset of the microsatellite markers included in a multiplex. Additionally, one individual showed heterozygosity for a specific marker that could not be reproduced in the second sample (Figure 9, Ujung). This variability is most likely attributable to the quality of the isolated DNA, as fecal samples may contain plant secondary compounds that can have a negative impact on the PCR process by interfering with the *Taq* polymerase enzymatic reaction (Marrero et al. 2009). In the cohort analyzed, samples from three individuals–Baru, Cisolok, and Hilo–showed amplification of seven out of eight microsatellite markers (Figure 9), highlighting the applicability of these microsatellites in gibbons and their potential for use in combination with fecal samples.

With the three examples described here, we illustrate the potential of the seven multiplexes for parentage analysis in apes and Old World monkey species other than macaques utilizing DNA not only isolated from blood, but also from hair follicle and potentially from fecal samples (Table 2).

**TABLE 2:** Summary of the applicability of the seven microsatellite‐based multiplexes across the species analyzed, including the type of DNA sample tested.

Species	Rhesus and long‐tailed macaque	Hamadryas baboon	Chimpanzee	Moloch gibbon
Type of DNA sample tested	Blood and hair	Blood	Blood and hair	Feces
MuA	Applicable	Applicable	Applicable (D11S925)	Potential
MuBC	Applicable	Applicable	Applicable (D3S1768)	Potential
MuD	Applicable	Applicable (D10S1412)	Applicable	N.D.
MuE	Applicable	Applicable (DXS2506)	Applicable (DXS2506)	N.D.
MuF1	Applicable	Applicable	^a^	N.D.
MuF2	Applicable	Applicable	^a^	N.D.
MHC‐STR set 2	Applicable	Applicable	Applicable (D6S291)	N.D.

*Note:* Microsatellite markers that lacked polymorphism in a specific species are indicated in brackets.

Abbreviation: N.D., not determined.

^a^ For the chimpanzee samples analyzed, MuF1 and MuF2 were combined into a single multiplex, MuF, at that time. In this combination, we observed that the markers MFGT27 and D5S820 did not amplify.

## 4 Discussion

The current study describes the development of seven microsatellite‐based multiplexes designed for parentage analysis, applicable to ape and Old World monkey species. The seven multiplexes include a total of 23 microsatellite markers distributed across 15 different chromosomes, adding 10 polymorphic markers across four additional chromosomes compared to the panels published by the INPRIMAT consortium. The seven multiplexes are thoroughly validated using a cohort of nearly 3000 individuals, consisting of pedigreed rhesus and long‐tailed macaque populations housed at the BPRC. In these populations, the 23 microsatellite markers within the multiplexes detect polymorphism, and the majority of the markers possess a PIC value of 0.5 or higher, indicating their high level of informativeness (Figure 3). Taken into account trio loci mismatching, parents could be assigned with either zero, one, two, or three mismatches in the well‐defined rhesus and long‐tailed macaque colonies at BPRC (Figure 4). Furthermore, our approach has proven to be instrumental in addressing a wide variety of parentage issues concerning questions related to inbreeding, stillborn animals, and true or false twin designations in our macaque colonies.

The 23 markers included in the seven multiplexes are generally sufficient to define parentage within a population. For our rhesus and long‐tailed macaque cohorts, where we have extensive background information on the animals and composition of breeding groups, we can achieve parentage determination even with three mismatches (Figure 4A). Our analysis revealed that most of these mismatches are due to allelic dropout at specific markers (Figure 4B). In some cases, however, true mismatches were identified between offspring and parents, but these did not affect parentage assignments. A few of these true mismatches may be attributed to mutations occurring between parent and offspring. In all cases where we assigned parents with more than one mismatch, we verified whether potential parents had been excluded from the analyses. In addition, in all such cases, the parents assigned based on the seven multiplexes were independently confirmed through MHC‐DRB‐STR and MHC‐A‐STR segregation (de Groot, Doxiadis, et al. 2008; Doxiadis et al. 2011). Overall, most of the 23 markers show no mismatches or only sporadic mismatches in a limited number of samples. However, on occasion we detected mismatches in a larger number of samples for specific markers, such as D2S169 in rhesus macaques and D5S820 in long‐tailed macaques (Figure 4B). These discrepancies are most likely due to technical inconsistencies, as these markers are successfully amplified at most analysis time points. Marker DXS2506 is the only exception, consistently displaying allelic dropout for a higher number of samples across all examined time points, particularly in long‐tailed macaque samples. Further improvement of parentage analyses in macaque species could involve integrating DXS2506 into a different multiplex or removing it from the panel. Overall, based on our experience with the species and populations examined, we emphasize that the current set of 23 markers spanning 15 chromosomes is sufficient for parentage determination, even in cases of inbreeding. However, we acknowledge the possibility that in some populations, the combination of markers in the seven multiplexes described may be insufficient to distinguish between closely related individuals, such as an alpha male and his sons. In such cases, incorporating additional microsatellite markers into the panel of seven multiplexes could improve parentage determination. Additionally, when applying the seven multiplexes to other non‐human primate species, such as Hamadryas baboons and chimpanzees, we observed that some microsatellite markers were polymorphic in one species but monomorphic in another. Although this affected only a few markers, and the remaining polymorphic markers were sufficient for parentage determination, there may be instances where replacing these markers could enhance the robustness of the multiplexes. While further testing may be required, a variety of established microsatellite markers have been documented for various primate species and could be integrated into the multiplexes as needed (Kanthaswamy et al. 2006; Newman et al. 2002; Raveendran et al. 2006).

The calculated parameters for the 23 different microsatellite markers showed higher average values for Ho and He in the long‐tailed macaque cohort (average Ho = 0.771 and He = 0.826) compared to the Indian rhesus macaque cohort (average Ho = 0.649 and He = 0.672). The increased genetic diversity in the long‐tailed macaque cohort is likely attributed to the introduction of 84 animals originating from the mainland north of the isthmus of Kra into the original breeding groups, which initially consisted of animals from the mainland south of the isthmus of Kra and the Indonesian and Malaysian islands (de Groot et al. 2022). This introduction was necessary to prevent inbreeding and maintain genetic diversity, thereby establishing the BPRCs long‐tailed macaque cohort seen as a mixed population. Indian rhesus macaques are known to have experienced a population decline caused by a bottleneck resulting from geographic changes (Hernandez et al. 2007; Smith and McDonough 2005), which might further explain the differences observed in the average values for Ho and He. For three microsatellite markers, D4S2365 in the Indian rhesus macaque, D7S794 in the long‐tailed macaque, and D5S1457 in both species, the observed heterozygosity was slightly higher than the expected heterozygosity (Figure 3B). This suggests that in these instances, the genetic diversity in the populations is somewhat greater than predicted based on the known genetic variation. Different factors could contribute to an increased Ho, such as genetic admixture, population structure, and an increase in the mutation rate (Amos et al. 2008; Boca et al. 2020). Among these, genetic admixture and population structure are the most likely contributors affecting the three microsatellite markers mentioned. The lowest Ho was observed for the X‐chromosome marker DXS2506 in both macaque cohorts (Figure 3B). The allele frequency distribution for this marker showed a bias towards the presence of allele 257 in the rhesus macaque cohort, whereas a more even distribution of alleles was observed in the long‐tailed macaques (Table S3). This low heterozygosity for the X‐linked marker and the bias towards allele 257 in the rhesus macaque cohort can be explained by the breeding strategy used at BPRC. Generally, rhesus macaque males born in our breeding cohort are selected to become alpha males. This potentially reduces the X‐linked diversity. X‐linked diversity might be enlarged in cases when alpha males from outside the cohort are selected. Additionally, the introduction of new females could potentially benefit X‐linked diversity. In the past 25 years, we only introduced six new alpha males and one new female residing outside the breeding colony, and their selection was primarily based on genetic variation in the MHC.

The introduction of parentage analysis at a molecular level for our macaque breeding groups revealed that successful pregnancies could result not only from copulation by the alpha male but also from sexual interactions involving young males within a group. These instances predominantly involved young females giving birth to the offspring. Given the higher chance of relatedness between these young males and females, we sometimes separate the young males already at an age of 3 years old. However, considering the low rates of inbreeding and the importance of maintaining the stability of a breeding group, it is preferable to keep young males in their natal group for a longer period. This extended stay allows them to gain valuable social experience that will be crucial for them when they eventually become an alpha male later in life. To prevent inbreeding, young females receive a contraceptive implant (Implanon) (Maaskant et al. 2024).

Parentage analysis in our rhesus and long‐tailed macaque colonies is routinely performed using DNA samples isolated from EDTA blood. In general, this blood‐derived DNA is of high quality, resulting in a good amplification of the microsatellite marker. In cases of stillborn rhesus and long‐tailed macaque animals, we receive hair follicle samples. The DNA isolated from these samples is generally of sufficient quality to allow for successful parentage determination. The effectiveness of the microsatellite multiplex on DNA isolated from hair follicles is further substantiated by the successful parentage determination of two newborn chimpanzees (Figure 8). In addition, we have explored the application of the seven multiplexes using DNA isolated from blood samples of Hamadryas baboons, while a subset of the multiplexes was tested for its amplification efficiency with fecal‐derived DNA samples from Moloch gibbons. These examples illustrate the applicability of the multiplexes for parentage analysis in other Old World monkey species besides macaques, as well as in lesser ape species. It also highlights the potential of using fecal samples, pending optimalisation of DNA isolation techniques and further studies evaluating the full set of seven multiplexes for parentage determination in these sample types.

The microsatellite‐based multiplexes rely on a traditional capillary electrophoresis‐based Sanger sequencing approach, which has its limitations (Guichoux et al. 2011; Lepais et al. 2020). For instance, when processing a high number of samples or during allele calling‐based on fragment length determination referenced to a size standard‐there can be inconsistencies in calls between different tests. A SNP‐based genotyping method referred to as genotyping by sequencing (GBS) takes advantage of next‐generation sequencing techniques, and is an approach that can help to address these limitations (Barbian et al. 2018; Salado et al. 2021; Sarhanova et al. 2018). GBS generates a large number of reads for multiple samples and microsatellite markers simultaneously. In addition, it produces interpretable sequences, for instance allowing for the discrimination of alleles with the same length that contain nucleotide polymorphisms. However, GBS also has its challenges. It is most (cost)efficient when analyzing a large number of samples and microsatellite loci, which also necessitates the use of advanced bioinformatic tools for data analysis (Darby et al. 2016; Salado et al. 2021). Various software tools are available nowadays, although improvements are still needed in accurate allele calling and addressing allele dropout (Flanagan and Jones 2019; Salado et al. 2021; Shestak et al. 2021). Optimization of sample quality can help circumvent these issues, as GBS is often applied in the context of fecal samples, which typically contain only small amounts of host DNA. Overall, capillary electrophoresis‐based Sanger sequencing with thoroughly characterized microsatellite panels has proven to be reliable and robust for parentage analysis. However, GBS, though not yet an established method, shows promising potential for future use.

In conclusion, we developed seven microsatellite‐based multiplexes that are applicable for determining parentage across apes and Old World monkey species and addressing inquiries regarding, for instance, the composition of breeding groups. In addition, the multiplexes are applicable not only to blood‐derived DNA, but also to DNA isolated from non‐invasively collected samples, such as hair follicle and potentially feces. The developed approach is suitable for monitoring parentage at a molecular level in conservation‐based studies conducted in zoo‐ and sanctuary‐housed as well as in free‐ranging non‐human primates. The latter might be in particular important for monitoring and potentially mitigating inbreeding depression in populations experiencing extreme declines in numbers, thereby aiding conservation strategies for these populations.

## Author Contributions

**Natasja G. de Groot:** data curation (equal), formal analysis (equal), investigation (equal), supervision (equal), writing – original draft (lead), writing – review and editing (lead). **Annemiek J. M. de Vos‐Rouweler:** data curation (equal), formal analysis (equal), investigation (equal), writing – original draft (equal). **Corrine M. C. Heijmans:** data curation (equal), formal analysis (equal), writing – original draft (equal). **Annet Louwerse:** writing – original draft (equal). **Jorg J. M. Massen:** writing – original draft (equal). **Jan A. M. Langermans:** writing – original draft (equal). **Ronald E. Bontrop:** writing – original draft (equal). **Jesse Bruijnesteijn:** writing – original draft (equal), writing – review and editing (equal).

## Conflicts of Interest

The authors declare no conflicts of interest.

## Supporting information


**Table S1.** Details of the microsatellite markers included in the seven multiplexes for parentage analysis. The table lists the chromosome (chr) location, primer sequence, primer concentration and labeling dye, and for each marker a reference. *Primers that are developed based on the *Macaca fuscata* genomic sequence; **Chromosome location based on the NCBI Map Viewer (http://www.ncbi.nlm.nih.gov/mapview/); ***Chromosome location based on mapping the primer sequences to Mmul_10 genome (GCF_003339765), which is an assembly of the rhesus macaque genome.

**Table S2.** (A) Total number of offspring per year (N) analyzed for parentage in rhesus and long‐tailed macaque samples, including the annual number of offspring for which the parents are assigned with 0, 1, 2, and 3 mismatches (mm) based on trio loci mismatching. The left panel displays data from both blood and hair samples, while the right panel shows data from the hair samples only. (B) Overview of the trio LOD score statistics per year for BPRC’s rhesus and long‐tailed macaque offspring, as well as for the Hamadryas baboon samples. For the rhesus and long‐tailed macaques, the total number of offspring per year is provided, along with the minimum and maximum LOD scores, mean LOD score, and standard (Std) error of the mean. For the Hamadryas baboon, the total number of animals analyzed is indicated, along with their LOD score statistics.

**Table S3.** Overview of the allele frequency (Freq) distribution of microsatellite markers in the seven multiplexes analyzed in BPRC’s rhesus and long‐tailed macaque cohorts. The frequencies were calculated using Cervus software. If an allele is not detected in the corresponding cohort, this is indicated with a “‐”.

**Table S4.** Parameters of the 23 microsatellite markers included in the seven multiplexes for the Hamadryas baboon cohort. The following data are provided for each microsatellite marker: number of alleles (k), number of typed animals (N), observed (*Ho*) and expected (*He*) heterozygosity, polymorphic information content (*PIC*), average non‐exclusion probability for one candidate parent (NE‐1P), average non‐exclusion probability for one candidate parent given the genotype of a known parent of the opposite sex (NE‐2P), significance of deviation from Hardy–Weinberg equilibrium (HW; NS not significant. ND not determined), and estimated null allele frequency (F(Null); ND not determined).

**Table S5.** Overview of the microsatellite length patterns detected for the two newborn chimpanzees Julian and Jozi (O), their respective mothers (M) and potential fathers (F1–F7). For each microsatellite marker in a multiplex, the fragment length of the two alleles is provided. When possible, segregation of the fragments is indicated by a colored background, with orange representing the maternal haplotype and light yellow the paternal haplotype. The microsatellite marker IDs that discriminate the fathers of Julian and Jozi are printed in red, and are the data included in Figure 8. If an allele is not detected, this is indicated with a “‐”. Family trees are included, with the analyzed individuals highlighted in yellow.

Data S1.

Data S2.

Data S3.

## Data Availability

The data that support the findings of this study are available as Data [Link], [Link], [Link].
